# EcoPhysioMechanics: Integrating Energetics and Biomechanics to Understand Fish Locomotion under Climate Change

**DOI:** 10.1093/icb/icac095

**Published:** 2022-06-27

**Authors:** Valentina Di Santo

**Affiliations:** Division of Functional Morphology, Department of Zoology, Stockholm University, Svante Arrhenius väg 18B, 11419 Stockholm, Sweden

## Abstract

Ecological physiologists and biomechanists have investigated swimming performance in a diversity of fishes; however, the connection between form, function, and energetics of locomotion has been rarely evaluated in the same system and under climate change scenarios. In this perspective, I argue that working within the framework of “EcoPhysioMechanics,” i.e. integrating energetics and biomechanics tools, to measure locomotor performance and behavior under different abiotic factors, improves our understanding of the mechanisms, limits and costs of movement. To demonstrate how EcoPhysioMechanics can be applied to locomotor studies, I outline how linking biomechanics and physiology allows us to understand how fishes may modulate their movement to achieve high speeds or reduce the costs of locomotion. I also discuss how the framework is necessary to quantify swimming capacity under climate change scenarios. Finally, I discuss current dearth of integrative studies and gaps in empirical datasets that are necessary to understand fish swimming under changing environments.

## Introduction

Over the past decades physiologists have suggested that investigating shifts in locomotor performance can be used to elucidate major mechanisms of organismal responses to climate change (Somero 2010; Lauder and Di Santo 2015; Lawson et al. 2019; Vilmar and Di Santo 2022). While linking climate data and ecophysiology has resulted in the establishment of the prolific field of “conservation physiology” (Wikelski and Cooke 2006; Cooke et al. 2013), biomechanics has yet to become integrated in many physiological studies, and it is rarely applied to work looking at locomotor performance under climate change scenarios (Helmuth et al. 2005; Denny and Helmuth 2009; Denny and Gaylord 2010; Carrington et al. 2015; Currier et al. 2021; Vilmar and Di Santo 2022). Successful integration has been slow mostly because physiologists and biomechanists generally focus on different aspects of locomotor performance (Breder 1926; Fry 1947; Johnson and Bennett 1995; Di Santo et al. 2021), and there is a lack of unifying frameworks to study mechanics and energetics of movement under a new interdisciplinary umbrella of “*EcoPhysioMechanics*.” Ecological physiologists typically quantify the effect of abiotic factors on performance such as, for example, oxygen consumption during locomotion or digestion (Fry 1947; Brett 1967; Roche et al. 2013; Bale et al. 2014; Deutsch et al. 2015), while biomechanists focus on the relationship between form and function to understand how organisms move under different physical conditions (Breder 1926; Lindsey 1978; Shadwick and Lauder 2006; Lauder 2015; Di Santo et al. 2021). Yet, the integration of these two well-established fields, ecophysiology and biomechanics, presents the opportunity to link movement and energetics of locomotion to understand plasticity and selection under environmental change.

**Fig. 1:**
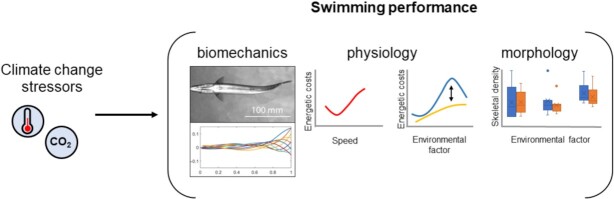
EcoPhysioMechanics framework. The consequences of environmental factors such as temperature and carbon dioxide (CO_2_) on individual and collective fish locomotion can be studied by integrating measurements from biomechanics (e.g., kinematics), physiology (e.g., performance curves, active metabolic rates), and morphology (e.g., density of skeleton). By combining ecophysiology and biomechanics, we can elucidate mechanisms underlying shifts in locomotor performance.

Here, I argue that integrating energetics and biomechanics studies to quantify locomotor performance under different abiotic conditions, including climate-related stressors, is key to understand organismal responses under stable, fluctuating, and changing environments, and the consequences of variation in swimming kinematics on physiological performance (Fig. 1). To illustrate how EcoPhysioMechanics can be applied to locomotor studies, I focus on a few cases that provide a thread across morphology, eco-physiology, and biomechanics in fishes. First, I outline how linking biomechanics and physiology of whole organisms can be used successfully to understand how fishes may modulate their kinematic behavior to achieve high speeds or to lower the costs of locomotion. Second, I discuss how this particular framework can be helpful to quantify swimming capacity in species under climate change scenarios. Finally, I conclude with a discussion of gaps in empirical datasets that are necessary to understand locomotion, and how the lack of integrative studies can hinder the progress of biomechanics, eco-physiology, and conservation biology.

## Integrating biomechanics and physiology to understand fish locomotor performance

Locomotor performance is a key contributor to the evolutionary success of fishes (Breder 1926; Hunter 1998). As a consequence, fish locomotion has been a major topic of investigation for functional morphologists, physiologists, and engineers (Breder 1926; Brett 1967; Lighthill 1971; Daniel 1984; Sfakiotakis et al. 1999; Bale et al. 2014; Lauder 2015; Di Santo et al. 2021; Akanyeti et al. 2022). Fishes display an extraordinary variety of body shapes and locomotor behaviors that they use to escape predators, attack prey, maneuver in complex habitats, perform large scale migrations, school, mate, communicate, and explore the substrate (Johnson and Bennett 1995; Wilga and Lauder 2002; Shubin et al. 2006; Clark 2016; Fox et al. 2018; Jung et al. 2018; Flowers et al. 2020). However, the dearth of integrative studies examining the energetic consequences and the limits of locomotor performance slows down our capacity to understand and forecast shifts in movement range and capacity, especially under environmental change (Helmuth et al. 2005; Vilmar and Di Santo 2022).

The important consideration with respect to unifying biomechanical and physiological studies in light of climate change is that the consequences of shifts in locomotor behavior resonate at the level of physiological processes, and as a result, changes in the environment can limit or expand the locomotor performance envelope (Johnson and Bennett 1995; Whitlow et al. 2019; Currier et al. 2021). Furthermore, individual locomotor decisions can alter collective behaviors (Couzin et al. 2005) and, as a consequence, population and ecosystem-level dynamics may change because schooling and shoaling are fundamental for the survival of forage fishes (Shaw 1962; Emmett and Sampson 2007; Saadat et al. 2021). Here, I analyze two sets of locomotor behaviors that show that the integration of biomechanics and physiology is important to understand the mechanisms that limit performance in solitary swimmers and schooling fishes.

**Fig. 2:**
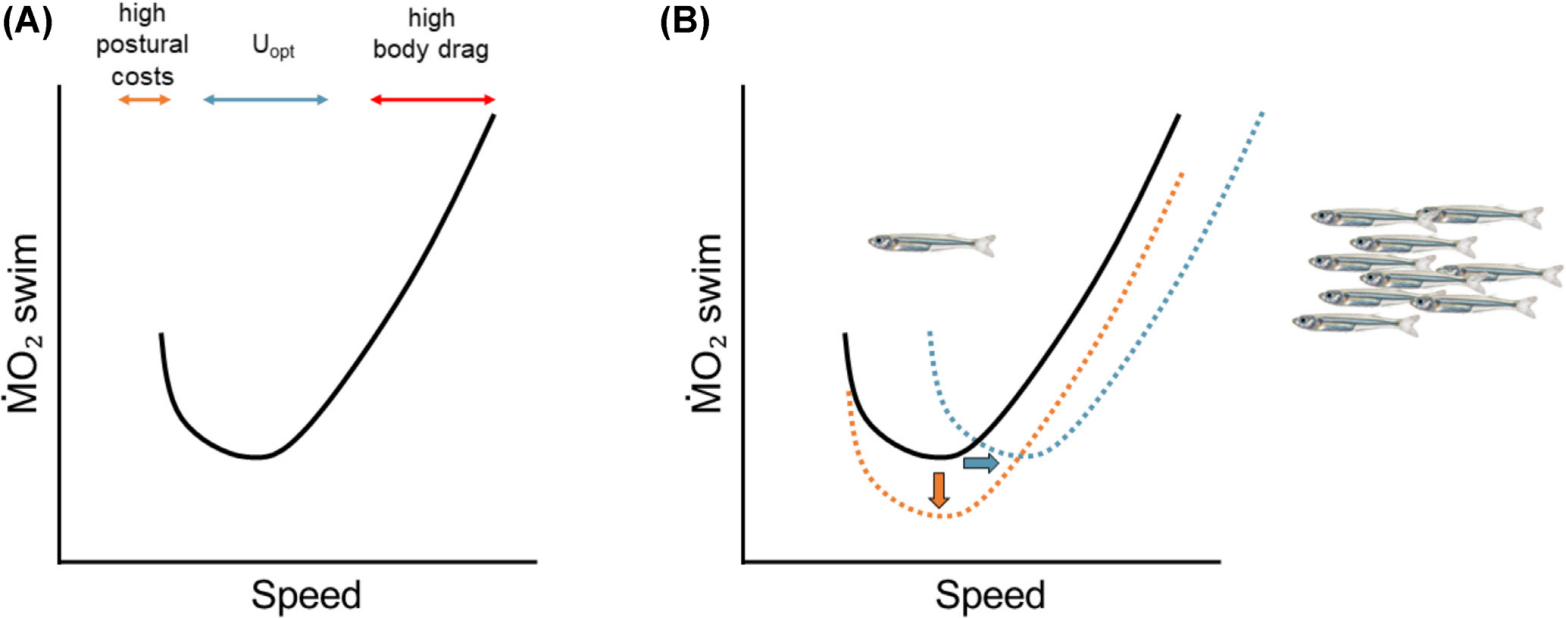
A J-shaped metabolic–speed relationship for swimming. **(A)** The relationship between metabolic rates (ṀO_2_) and speed is predicted to be J- or U-shaped because fishes may experience high postural costs at low speeds, and high drag as speed increases. We should expect to find an intermediate optimal speed (*U*_opt_) at which swimming is relatively economical. **(B)** Fishes may take advantage of a group formation during schooling to reduce the costs of swimming (curve shifts down, orange dotted line) or to swim faster more efficiently (*U*_opt_ shifts towards the right, teal dotted line).

Hydrodynamics models have predicted that the relationship between speed and metabolic rates should follow a U- or J-shape (Webb 1994; Sepulveda et al. 2003; Di Santo and Kenaley 2016; Di Santo et al. 2017b). In fact, energetic costs are expected to increase at low speeds as postural costs and induced drag become significantly elevated and at high speeds as body drag increases with speed (Vogel 2020). Therefore, there should be some intermediate speeds at which swimming is relatively economical, i.e. the optimal speed or *U*_opt_ (Fig. 2). However, many studies in the past decades showed a linear or exponential metabolic–speed curve (some examples: Webb 1994; Lauder and Di Santo 2015) when data at low speeds were either eliminated or possibly ignored (Sepulveda et al. 2003, 2007). Many data sets show extrapolation of resting metabolic rates at speed = 0 from swimming data, which can be significantly different from empirically obtained rates (Lee et al. 2003). The reason is that extrapolation from swimming data ignores the elevated postural costs during hovering when compared to resting, and in fact some fishes may allow extreme rolling of their body to save energy during resting periods (Ciancio et al. 2016). Even fishes with a swim bladder may need to continuously move their fins to avoid rolling during hovering, and the energy used to execute these movements should be higher than simply resting (Priede and Holliday 1980; Duthie 1982; Lauder and Madden 2007; Di Santo et al. 2017b). Several researchers disclosed in their papers that the lowest speed for swimming experiments (usually around 1 BL/s) was selected based on the fact that at velocities below 1 BL/s the fish would swim erratically (Sepulveda et al. 2003, 2007; Behrens et al. 2006). More work focusing on the energetics and biomechanics of hovering in a wide range of negatively and nearly neutrally buoyant fishes may improve our understanding of the postural costs of “swimming in place.” Studies on skates (little skate *Leucoraja erinacea*, and clearnose skate *Raja eglanteria*) combined physiological and biomechanical measurements to answer the question of whether energetics and postural issues may shape and limit swimming performance (Di Santo and Kenaley 2016; Di Santo et al. 2017a; Di Santo et al. 2017b). These studies show that at low speeds (<1 BL/s) fishes significantly increase oxygen consumption during swimming when compared to an intermediate *U*_opt_ (∼1.25–1.5 BL/s) and that these increased energetic costs are attributable to high postural costs to maintain equilibrium and to a significant anaerobic component of metabolism during steady swimming that is often ignored in energetics studies (Di Santo and Kenaley 2016; Di Santo et al. 2017b). Even though negative buoyant fishes, such as elasmobranchs, may experience high costs of locomotion at low speeds due to the necessity of moving their fins to produce hydrodynamic lift, data on rainbow trout (*Oncorhynchus mykiss*) show that postural costs at low speeds can be detected in fishes with a swim bladder as well (Di Santo et al. 2017b). At 0.5 BL/s trout assume a positive body angle to the flow and use their dorsal fin to control body stability, while at 1.25 BL/s they swim straight into the flow and fold down their dorsal fin (Di Santo et al. 2017b). As a consequence, metabolic rates at 0.5 BL/s are higher than at 1.25 BL/s and the metabolic–speed curve in trout is J-shaped (Di Santo et al. 2017b).

Kinematics of swimming influence the costs of locomotion, but physiological processes can, in turn, affect movement. Several studies have shown that the ratio of lactate produced:disposed increases at intermediate-to-high speeds (Weber 1991; Peake and Farrell 2004; Svendsen et al. 2010). The increase of lactate in body fluids and tissues limits the swimming performance of fishes (Black et al. 1962; Jain et al. 1998; Kieffer 2000; Jain and Farrell 2003; Widmer et al. 2006). In addition, upper sustained speeds might be limited by the body posture and fin movements that are necessary to create thrust. One example is the undulatory movement of the pectoral fins in batoid fish at high speeds. The upper speed limit for benthic batoids, such as skates, seems to be around 2 BL/s (Rosenberger and Westneat 1999; Rosenberger 2001; Di Santo et al. 2017a). When tridimensional kinematics of skate swimming are analyzed, it is apparent that skates’ upper velocity is limited by the energy spent by actively stiffening the pectoral fins to create a notch, or an arc, that travels from anterior to posterior across the fin margin. Such notch is only noticeable at the maximum sustainable speed for the fish, suggesting that the limit to benthic batoid locomotion may be also biomechanical rather than just physiological (e.g., the use of anaerobic metabolism) (Di Santo et al. 2017a).

Schooling behavior is considered fundamental to the survival of the great majority of fishes, and especially of forage species (Shaw 1960, 1961, 1962; Couzin and Krause 2003; Jolles et al. 2017). In fact, schooling behavior favors the detection of food and mates, reduces the risk of predation, and may increase locomotor efficiency (Weihs 1973; Herskin and Steffensen 1998; Ward and Webster 2016; Papastamatiou et al. 2021). Fish in schooling formations display extraordinary swimming coordination, where evenly spaced individuals move in the same direction and assume parallel positions (Shaw 1962; Weihs 1973; Katz et al. 2011; Ashraf et al. 2017; Kent et al. 2019). Models suggest that fish maintain a relatively stable distance among individuals and it is exactly this spacing and how it changes under different flow and abiotic conditions that determine the hydrodynamic effects of individuals swimming in the school (Weihs 1973; Kent et al. 2019). According to theoretical models, fish may gain a hydrodynamic advantage by positioning themselves in a diamond configuration within the aggregation due to the pattern of vortex trails formed by neighboring swimming fish (Weihs 1973). Directly behind a swimming fish, the vortex trail has increased water velocity opposite to the swimming direction but with the slight lateral shift, fish in trailing positions can benefit of increased velocity in the same direction as the school is swimming, saving energy associated with locomotion (Weihs 1973). At the same time, recent work showed that also phalanx, where fish swim side by-side and synchronize their tail beat, and in-line configurations may correlate with reduced tail beat frequency, a proxy for metabolic expenditure (Ashraf et al. 2017; Saadat et al. 2021). However, very few studies to date measured the energetic costs of swimming in a school (Burgerhout et al. 2013; Currier et al. 2021). Energetic costs of schooling have been difficult to quantify because it is challenging to separate the energetics of locomotion between individuals swimming in the front and periphery of the school from those swimming behind other fish. Individuals in a formation may change position, and consequently, the whole school may have significantly different metabolic rates at the same speeds when assuming different geometric configurations. Although individual metabolic rates are important, more studies should focus on the school (or group swimming, including pair and shoaling) as the “*unit*” to quantify the effect of collective movement on energetics. Currier et al. (2021 ) quantified the effect of group size on metabolic rates and tail beat frequency in bluegill sunfish (*Lepomis macrochirus*) and rainbow trout. When bluegill sunfish swim in groups their metabolic rates and tail beat frequency decrease, while the opposite effect has been quantified in trout across speeds (Currier et al. 2021). Unlike forage fishes, trout and bluegill sunfish do not strictly school, but it is possible that bluegill sunfish may take advantage of the group formation by decreasing the interindividual distance as seen in other species, such as zebrafish (*Danio rerio*) and rainbowfish (Family: Melanotaenia) (Wiwchar et al. 2018; Kent et al. 2019). I can therefore imagine that future studies may consider the role of the shape and volume of the formation on swimming aerobic performance and biomechanics. For instance, can fishes increase their *U*_opt_ by swimming in a school? Do schools reduce the metabolic rates of individual fish with no increase in *U*_opt_ (Fig. 2)? Preliminary work suggests that swimming in a school could increase the optimal swimming speed in Inland silverside *Menidia beryllina* (Di Santo and Lauder 2019, 2021). When oxygen consumption rates were measured in a small school (n = 3 individuals per school) of Barents Sea capelins (*Mallotus villosus*), fish showed difficulties swimming at speeds below 1 BL/s (Behrens et al. 2006). This detail not only suggests that low speeds may be difficult to test, but also that there might be a minimum number of individuals in a school that is needed to provide the energetic advantage of group swimming (Li et al. 2019; Currier et al. 2021). Correlations between tail beat frequency and metabolic rates can provide a good proxy for energetic expenditure and may be used instead of oxygen consumption measurements when these are not feasible (Herskin and Steffensen 1998; Lowe 2001; Ohlberger et al. 2007); however, not all fish species exhibit a linear relationship between metabolic rates and tail beat frequency (Leonard et al. 2000; Di Santo et al. 2017b; Currier et al. 2021), thus quantifying both measurements of energy consumption and tail beat frequency is preferred.

These two examples show that linking biomechanics and energetics allows to answer fundamental questions in animal locomotion such as which factors limit minimum and maximum sustainable speeds, which tactics can organisms employ to expand their performance envelope, and which consequences locomotor movements have on the energetic budget and behavior of organisms (Fig. 1).

## Climate change stressors alter fish locomotor performance

Recent anthropogenic activity has resulted in the exponential increase in greenhouse gases (in particular, carbon dioxide or CO_2_, methane or CH_4_) that has caused the oceans to become warmer and more acidic (Doney et al. 2009; Gattuso and Hansson 2011; Collins et al. 2018; Sadhukhan et al. 2020). The effects of ocean warming have already been vastly investigated on performance and behavior of fishes, with studies on ocean acidification following behind (Belkin 2009; Ryu et al. 2018; Clark et al. 2020). Studies on the effect of temperature on metabolic rates have proliferated as temperature, considered the “abiotic master factor”, has profound effects on fish physiology (Fry 1967; Angilletta 2009). In fact, nearly every physiological process is affected by temperature, and it is not surprising therefore, that fishes may use temperature as an ecological resource by exploiting thermal gradients to enhance physiological performance (Di Santo and Bennett 2011a; Krehl and Soetbeer 1899; Fry 1967; Magnuson et al. 1979; Wardle 1980; Jain and Farrell 2003; DiGirolamo et al. 2012).

Warming can enhance locomotor performance because muscle efficiency increases with temperature (Di Santo and Bennett 2011b; Johnston et al. 1990; Sims et al. 2006; DiGirolamo et al. 2012). For instance, fishes might reduce contraction times at higher temperatures, thereby increasing speed during burst swimming even when maintaining the same stride length (Wardle 1980; Wardle et al. 1995). This process might at least be partially responsible for the high performance of fast swimming species such as barracudas and tunas (Wardle 1980). Several studies have now demonstrated that warming can enhance escape responses in teleost fishes (Johnston et al. 1991; Wilson et al. 2001; Fernández et al. 2002; Lyon et al. 2008). However, myotomes may have limited capacity to adjust to changes in temperature, and locomotor performance may show no compensation with acclimation (Coughlin et al. 2020). The effect of warming on escape performance has been tested on one elasmobranch, the little skate (Di Santo 2016). In this study, skates showed local adaptation in temperature-performance curves. Skates from two neighboring locations (O'Connell et al. 2019) exhibit different thermal optima for endurance and number of bursts, with one population performing better under warming of 3°C when compared to currently experienced temperatures (Di Santo 2016). However, both populations show a decline in intensity of bursts and prolonged recovery time with 5°C warming suggesting that high power movements may become limited at temperatures expected by the end of the century (IPCC 2014; Di Santo 2016; Pinsky et al. 2019). It is also possible that the biomechanical advantage given by higher muscle contractility may not necessarily translate into higher escape performance; however, warming can affect other morphological structures, such as an increase in number of vertebrae which can produce greater linear displacements and higher speeds during escape responses (Ackerly and Ward 2016).

Temperature may reduce endurance and increase the costs of steady swimming across speeds (Brett 1967; Steinhausen et al. 2008; McDonnell and Chapman 2016). As warming increases the costs of locomotion of individual fish, we expect that fish swimming in a school may employ tactics to reduce these costs. However, schooling fishes show a lower degree of cohesiveness and polarization with warming (Bartolini et al. 2015; Davis et al. 2019). It is unclear though if the subsequent increase in tail beat frequency is the direct consequence of elevated temperatures or rather a side effect of looser aggregations and the inability to capture the vortices shed by neighboring fish (Weihs 1973; Ashraf et al. 2017; Saadat et al. 2021). If warming causes the disruption or loosening of the school, then the hydrodynamic advantages of swimming in a school formation may be lost.

Ocean acidification, the “other CO_2_ problem” (Doney et al. 2009) has a complex effect on fish morphology, locomotion, and behavior (Bignami et al. 2013; Di Santo, 2015, 2019; Clark et al. 2020). For instance, high CO_2_ levels prolong the time to recover from a chasing event (Di Santo 2016) and can decrease the maximum speed reached during swimming (Watson et al. 2018). High CO_2_ also increases the energetic costs of burst swimming while decreasing the rate of bursts (Di Santo 2016; Rummer et al. 2020). This suggests that an increase in metabolic rates during activity should not necessarily be interpreted as a positive outcome when the increase in energy spent does not translate into higher efficacy (endurance, rate of movement, speed, etc.) of locomotor behavior (Di Santo 2015; Lefevre 2016). The increase in CO_2_ has been associated with larger otoliths (Checkley et al. 2009; Bignami et al. 2013; Kwan and Tresguerres 2022) and higher density of the skeleton of marine fishes both in the lab and under natural settings, for instance near CO_2_ seeps (Di Santo 2019; Mirasole et al. 2020). A denser, heavier skeleton may result in higher costs of swimming (Drucker and Lauder 2000; Wilga and Lauder 2002; Di Santo 2019). However, other locomotor behaviors such as walking on the substratum might be enhanced by denser and stronger “walking fins” allowing more stable and effective benthic movement (Lucifora and Vassallo 2002; Standen et al. 2014; Di Santo 2019). Ocean acidification is also known to corrode denticles of sharks (Dziergwa et al. 2019), thus potentially reducing the locomotor advantages provided by a non-smooth body surface (Domel et al. 2018; Muthuramalingam et al. 2019).

On the other hand, fishes can fully compensate for CO_2_-induced respiratory acidosis and restore blood pH by flux of H^+^ and HCO_3_^−^ using their gills (Claiborne and Evans 1992; Claiborne et al. 2002; Damsgaard et al. 2015; Wright and Wood 2015; Brauner et al. 2019; Kwan and Tresguerres 2022). The rate and to what extent acid–base compensation is reached when fishes are exposed to elevated CO_2_ depends on a variety of factors, including water ion composition and the upper limit of increase in plasma (Brauner et al. 2019). Many fishes that naturally live under high or fluctuating CO_2_ conditions seem to possess an exceptional capacity for intracellular pH regulation, and it is therefore not surprising that ocean acidification effects might be less significant in such fish populations (Couturier et al. 2013; Heinrich et al. 2016; Rosa et al. 2017; Clark et al. 2020). For instance, the swimming performance of Atlantic cod (*Gadus morhua*) larvae is largely unaffected by ocean acidification (Maneja et al. 2013, 2015). Atlantic silverside (*Menidia menidia*) experience high seasonal fluctuations in pH and embryos developing later in the season under high CO_2_ are the least sensitive to ocean acidification (Baumann et al. 2018; Murray and Baumann 2018; Baumann 2019). Escape response in larval yellowtail kingfish (*Seriola lalandi*) was affected to a greater degree by warming than acidification (Watson et al. 2018). In fact, CO_2_ had no significant effect on the latency of reaction to startle stimuli or maximum speed in kingfish (Watson et al. 2018). Although physiological mechanisms of acid–base control are well studied, we still lack large body of evidence linking acidosis compensation and swimming mechanics and performance under ocean acidification.

## The future of EcoPhysioMechanics

EcoPhysioMechanics studies offer the opportunity to understand the consequences of changes in the environment on swimming mechanics and energetics. Movement and energetics are intimately connected and quantifying the costs of different locomotor behaviors and the biomechanics of movement across a range of environmental factors can provide a much clearer picture of the limits of performance and acclimation potential. Working within a unifying approach that integrates the fields of eco-physiology and biomechanics produces a framework that benefits the work of not only physiologists and biomechanists, but also of climate change and conservation biologists, ecologists, and engineers as these can predict locomotor performance and its consequences under different conditions (Fig. 1). Progress towards this framework requires that more studies combine measurements of morphology, kinematics, and energetics of fish swimming under different abiotic conditions, in the lab as well as in the wild (Long Jr et al. 2010; Lauder and Di Santo 2015; Porter et al. 2020; Lauer et al. 2022). This approach will favor the identification of physiotypes and morphotypes that might be vulnerable or resilient to rapid changes in the environment (Somero 2010; Byrne and Przeslawski 2013; Couturier et al. 2013; Vilmar and Di Santo 2022).

## Funding

Participation to the symposium was supported by The Company of Biologists and The Swedish Research Council (#2021-04400).

## Data Availability

Data discussed in this perspective are available from the authors and the original papers.

## Notes

From the symposium “Integrating ecology and biomechanics to investigate patterns of phenotypic diversity: Evolution, development, and functional traits” presented at the annual meeting of the Society for Integrative and Comparative Biology virtual annual meeting, January 3–February 28, 2022.
